# Lakes-scale pattern of eukaryotic phytoplankton diversity and assembly process shaped by electrical conductivity in central Qinghai-Tibet Plateau

**DOI:** 10.1093/femsec/fiad163

**Published:** 2023-12-14

**Authors:** Huan Zhu, Xiong Xiong, Benwen Liu, Guoxiang Liu

**Affiliations:** Institute of Hydrobiology, Chinese Academy of Sciences, Wuhan 430072, China; Institute of Hydrobiology, Chinese Academy of Sciences, Wuhan 430072, China; Institute of Hydrobiology, Chinese Academy of Sciences, Wuhan 430072, China; Institute of Hydrobiology, Chinese Academy of Sciences, Wuhan 430072, China

**Keywords:** algal indicator, Changtang endoheric region, climate change, phytoplankton community, threshold

## Abstract

Phytoplankton are the main primary producers in aquatic ecosystems and play an important role in food web and geochemical cycles. Its diversity, community structure, and assembly process are influenced by several factors. Alpine lake ecosystems are relatively weak and extremely sensitive to global climate change. However, the impact of climate change on phytoplankton in Qinghai-Tibet Plateau lakes and their responses are still unclear. In this study, we analyzed the diversity, environmental drivers, and assembly process of phytoplankton community in the central QTP lakes. The phytoplankton of these lakes can be primarily distinguished into freshwater and brackish types, with significant differences in species diversity and community dissimilarity. Both shared nearly same key environmental factors that significantly affecting phytoplankton such as EC, and brackish lakes were also positively correlative with TN. Stochastic process was predominant in phytoplankton assembly. Additionally, freshwater and brackish lakes were dominated by dispersal limitation and heterogeneous selection respectively. Alpine lakes had significant EC thresholds, and their diversity and assembly processes changed significantly around the thresholds. The present findings have important implications for understanding and predicting the response of lake phytoplankton communities to climate change and for making decisions to protect the ecological resources of alpine lakes.

## Teaser

The variations in the phytoplankton diversity and assembly process in Central Tibetan Plateau lakes along Electrical Conductivity.

## Introduction

Eukaryotic phytoplankton are a complex group of diverse microorganisms widely scattered in various aquatic habitats, and are the fundamental feature of aquatic systems, which can also be used to characterize water quality (Soballe and Kimmel 1987). Although eukaryotic phytoplankton play a variety of key ecosystem roles as important primary producers, they can also occasionally cause harmful algal blooms under certain conditions (Tranvik et al. 2009, Borics et al. 2021). A large number of studies have focused on the dynamics of phytoplankton communities and their environmental drivers, and the abundance, structure, and assembly of these phytoplankton communities are known to be sensitive to climate-mediated physical forces and changes in nutrient enrichment (Winder and Sommer 2012, Klais et al. 2017, Borics et al. 2021, David et al. 2021). However, only a few studies have been performed on phytoplankton communities in alpine lakes, which are considered to be sensitive indicators of climate change (Elser et al. 2020). Recently, studies focused on alpine freshwater lakes have reported that changes in phytoplankton communities may be driven by nitrogen and phosphorus deposition (Wolfe et al. 2003, Tolotti et al. 2006, Brahney et al. 2015). In peri-alpine lakes, phytoplankton community is mainly driven by factors that are strongly influenced by climate change and eutrophication (Anneville et al. 2005, Gallina et al. 2013). In brackish alpine lakes, salinity is the critical driving factor regulating both bacterial and microeukaryotic communities (Liu et al. 2020). The shifts in salinity and other nutrients caused by expansion or shrinkage of these lakes and human activities can strongly contribute to changes in the plankton communities and gross primary productivity (Jia et al. 2021, Li et al. 2021, Liang et al. 2021). Overall, climate change, associated with changes in hydrological condition and nutrition shifts, is considered to be one of the possible reasons for phytoplankton community changes in alpine lakes. Many studies have demonstrated that the mechanistic links between climate change and phytoplankton dynamics are very important to assess the impacts of climate change on aquatic ecosystems (Winder and Sommer 2012); however, research on phytoplankton communities in Qinghai-Tibet (Xizang) Plateau (QTP) lakes is limited.

It has been reported that the phytoplankton communities in QTP lakes have a low alpha diversity and high beta diversity owing to the effects of harsh environmental conditions (Yang et al. 2018). The changes in both lake salinity and water temperature have been reported to significantly affect the trophic structure, trophic interactions, and biodiversity in aquatic ecosystems of lakes around Siling Co (Zhu et al. 2019). Another key issue in microbial ecology is to quantify the ratio of deterministic niche processes and stochastic neutral processes in microbial community assembly (Stegen et al. 2013, Dini-Andreote et al. 2015). The debate on microbial community assembly process has been long and mainly focused on the coexistence of niche processes and neutral processes, and previous studies have shown that there is no general consensus on the process of phytoplankton assembly (Zhou et al. 2014). In general, the relative contributions of both the assembly processes are related to environmental variables (Dini-Andreote et al. 2015, Feng et al. 2018). Phytoplankton have distinct abundance, and studies on the effects of ecological processes on the phytoplankton community structure and environmental variables gradient in alpine lakes, especially QTP lakes, are scarce.

In the past three decades, QTP experienced evident climate changes and showed overall surface warming and moistening (Kang et al. 2010, Yao et al. 2019). Such significant warming and humidification are very critical to the ecological systems across the plateau, including the aquatic ecosystems. Many lake ecosystems have experienced long-term changes as a result of other ecological pressures introduced by climate changes that could affect phenology. There is strong evidence indicating that climate change has a significant impact on the reproductive phenology of QTP fish as well as other species in terrestrial ecosystems (Zhuang et al. 2010, Tao et al. 2018); however, their impact on phytoplankton in QTP lakes as well as the responses of these phytoplankton are still unclear. The hydrological changes in adjacent but unconnected lakes can be used to infer phytoplankton community dissimilarity, environmental drivers, as well as assembly processes, and are crucial to determine the outcome of environmental changes. The Changtang endorheic region is the central part of the QTP, which is the largest uninhabited area far from human activities and with numerous lakes in China. We considered these lakes with a certain gradient in terms of various environmental factors as an ideal model to investigate phytoplankton diversity and their environmental drivers, and thus further study the effects of climate change on aquatic ecosystems, especially phytoplankton.

In recent years, the Uthermoll sedimentation method and microscopic observation for the identification of algae has presented many challenges owing to the phenotypic plasticity and cryptic diversity of algae (Leliaert et al. 2014, Verbruggen 2014). Metabarcoding sequencing can better avoid these deficiencies caused by traditional microscopic identification (Blaxter 2004), although this method also has some limitations, such as variation in gene copy number (GCN) between different species (Angly et al. 2014). With regard to QTP lakes, lakes-scale patterns of molecular diversity and assembly process of eukaryotic phytoplankton still remain poorly profiled, despite their importance in understanding alpine lake productivity. Furthermore, studies on the diversity and ecology of QTP are largely focused on terrestrial environments, and those on aquatic ecosystem are mainly aimed at fishes or bacteria. Owing to the limited research on the molecular diversity and ecology of phytoplankton, the applicability of ecological theories obtained from metazoans, embryophytes, or bacteria still remain unclear for eukaryotic microorganisms. Accordingly, the primary aims of present study were: (1) to comprehensively profile the diversity and assembly process of eukaryotic phytoplankton community in those ultra-alpine lakes, and reveal the key driving factors; (2) to assess impacts of climate change induced decrease in electrical conductivity on phytoplankton diversity in various Qinghai-Tibet Plateau lakes, and thus to predict how phytoplankton diversity patterns will change with fluctuations in electrical conductivity across different alpine lakes.

## Materials and methods

### Sampling and evaluation of environmental variables

**Figure 1.**
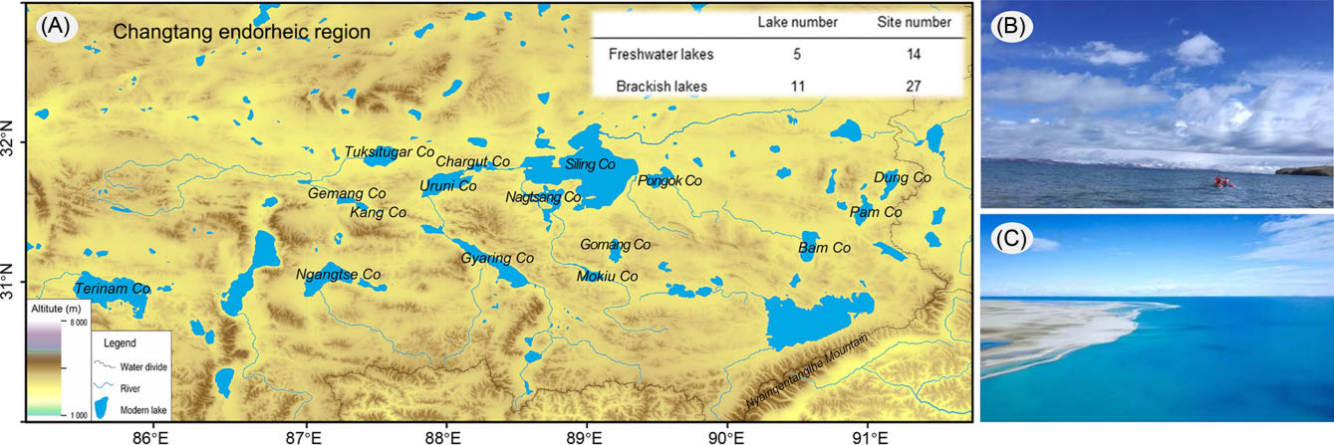
Map of the studied areas in central QTP and the aerial view of two typical lake groups. (A) Locations of 16 lakes in Changtang endorheic region. (B) Representative freshwater lake, Gyaring Co. (C) Representative brackish lake, Siling Co.

A total of 16 lakes, including 5 freshwater lakes and 11 brackish lakes, in the central Changtang endorheic region were selected (Fig. 1). At least two samples from different pelagic zones in the same lake were collected from May to July 2015. The altitude and coordinates were obtained using a GPS tracker (Garmin oregon 750), and detailed sampling information is provided in Table S1. Water temperature, salinity, electrical conductivity (EC), pH, total dissolved solids (TDS), and dissolved oxygen concentration (DO) were measured in field using a Hydrolab Hash (Austin, TX, USA). The concentrations of total nitrogen (TN), ammonium aitrogen (NH_4_-N), nitrate nitrogen (NO_3_-N), nitrite nitrogen (NO_2_-N), total phosphorus (TP), soluble reactive phosphate (SRP), and other biogenic elements and heavy metal ions were determined as described previously (Xiong et al. 2020). For environmental DNA extraction, 500 ml of the collected samples were immediately filtered through 0.22-µm (in diameter) Durapore membranes (Millipore) using a peristaltic pump, and all the membranes were instantaneously placed into liquid nitrogen and stored.

### Sequencing and annotation of metabarcoding data

The DNA samples were processed using OMEGA Water DNA Kit according to the manufacturer's instructions. The universal primer (F: 5′-CCAGCASCYGCGGTAATTCC-3′; R: 5′-ACTTTCGTTCTTGATYRA-3′) was used to amplify the SSU rRNA V4 region from the genomic DNA extracted from each sample (Stoeck et al. 2010). The total volume of the reaction mixture for PCR was 10 μL, and the PCR conditions were as follows: initial denaturation at 95 °C for 5 min; 25 cycles of denaturation at 95°C for 30 s, annealing at 50°C for 30 s, and extension at 72°C for 40 s; and a final step at 72°C for 7 min. The PCR amplicons were purified using Agencourt AMPure XP Beads (Beckman Coulter, Indianapolis, IN, USA) and quantified using Qubit dsDNA HS Assay Kit and Qubit 4.0 Fluorometer (Invitrogen, Thermo Fisher Scientific, Oregon, USA). Subsequently, the amplicons were pooled into equal amounts. For sequencing and construction of library, Illumina HiSeq 2500 was used, and the raw data of all the samples have been deposited in the SRA of the NCBI database under the accession number, PRJNA865087. The raw data were primarily filtered and merged, and removal of primer base pairs was performed using USEARCH (Edgar and Flyvbjerg 2015). The high-quality reads generated from the above-mentioned steps were used in the following analysis. Sequences with similarity ≥ 97% were clustered into the same operational taxonomic unit (OTU) by USEARCH (v10.0), and OTUs with abundance <0.005% were filtered, followed by chimera removal using UCHIME (version 8.1) (Edgar et al. 2011). The representative sequences of each OTU were annotated by USEARCH (v10.0), and only eukaryotic algal OTUs were subsequently processed (Edgar 2010).

### Statistical analysis

The sequence matrix was aligned using Mafft online service (Katoh et al. 2019), and then trimmed using TrimAl (Capella-Gutierrez et al. 2009). Phylogenetic tree was constructed using IQtree (Lam-Tung et al. 2015), and the obtained consensus tree was used for calculating phylogenetic diversity and assembly process. To explore the differences in phytoplankton community patterns, principal coordinate analysis (PCoA) and non-metric multidimensional scaling (NMDS) clustering based on Bray-Curtis distance were performed. The richness, Shannon–Wiener diversity, and phylogenetic diversity indices were used to measure alpha diversity, while the community dissimilarity (based on Bray–Curtis, Jaccard, and Morisita distances) was calculated to determine beta diversity. All these indices were computed and compared using VEGAN and PICANTE packages (Kembel et al. 2010, Oksanen et al. 2017). To identify the key environmental factors, redundancy analysis (RDA), variance partitioning analysis (VPA), and Mantel test were performed using VEGAN packages. To explore the mechanism of community assembly in different lake groups, the ecological processes affecting phytoplankton assembly were quantified using Picante, iCAMP, and NST packages (Stegen et al. 2013). The positive (z+) and negative (z−) responding OTUs (threshold indicator taxa analysis, TITAN) for both brackish and freshwater lakes were identified, and the change points of phytoplankton community in response to key environmental variables (change-point analysis, nCPA) were detected using TITAN2 (Baker and King 2010). All numerical ecological analyses were performed in R (R Core Team 2021).

## Results

### Comparison of phytoplankton diversity in brackish and freshwater lakes

**Figure 2.**
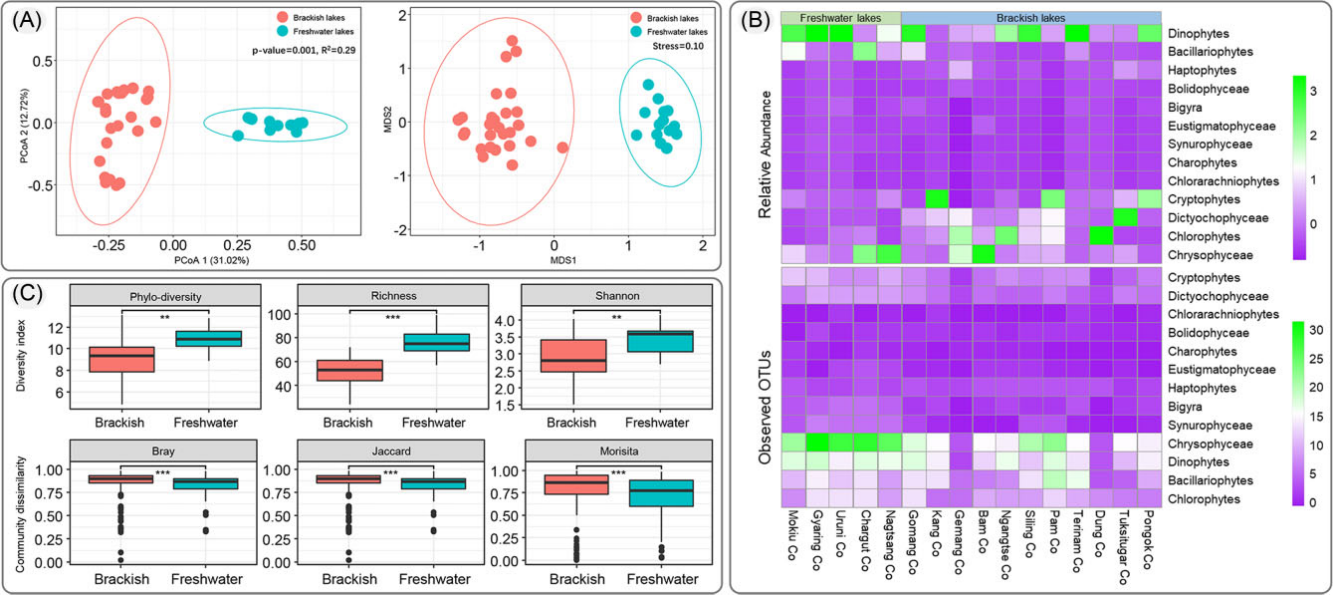
Community structure of eukaryotic phytoplankton across Changtang endorheic region lakes and comparison of their assembly, diversity, and community dissimilarity. (A) Grouping of phytoplankton communities according to compositional similarity (Bray–Curtis distance) using PCoA (adonis analysis r^2^=0.29, *P*=0.001) and NMDS (stress=0.10), respectively. Red and blue dots represent brackish and freshwater lakes, respectively. (B) Heatmaps indicating the relative abundance (z-value) and number of observed OTUs of phytoplankton composition across all the studied lakes. (C) Comparison of species diversity (including phylogenetic diversity, richness, and Shannon–Wiener diversity indices) and community dissimilarity (including Bray–Curtis, Jaccard, and Morisita distances) between brackish and freshwater phytoplankton communities.

The obtained clean reads from 16 lakes were clustered into 449 OTUs, of which 185 OTUs were annotated as eukaryotic phytoplankton belonging to 8 phyla (Tables S2–S3; Fig. S1). The detailed information, including closest species, GenBank accession number, and % identity of each phytoplankton OTU is provided in Table S4. The plateaued rarefaction graph indicated that the data are adequate for further analysis (Fig. S2). Both PCoA and NMDS ordinations clearly showed that the phytoplankton communities separately grouped into brackish lakes and freshwater lakes (Fig. 2A). Dinophytes and Chrysophytes were dominant with respect to relative abundance and number of observed OTUs, respectively (Fig. 2B). Overall, the abundance of Bacillariophytes in freshwater lakes was higher than that in brackish lakes, whereas the abundances of Dictyochophytes and Chlorophytes in freshwater lakes were lower than those in brackish lakes. The phylogenetic diversity and alpha diversity of phytoplankton communities in freshwater lakes were significantly higher than those of phytoplankton communities in brackish lakes (Fig. 2C). However, community dissimilarities based on three distances of freshwater lakes were significantly lower than those in brackish lakes (Fig. 2C). Based on these results, the phytoplankton communities in QTP lakes could be primarily classified into two types according to salinity or EC, namely, brackish and freshwater phytoplankton communities. Subsequently, these two types of phytoplankton communities were respectively analyzed.

### Correlation between phytoplankton diversity and environmental variables

**Figure 3.**
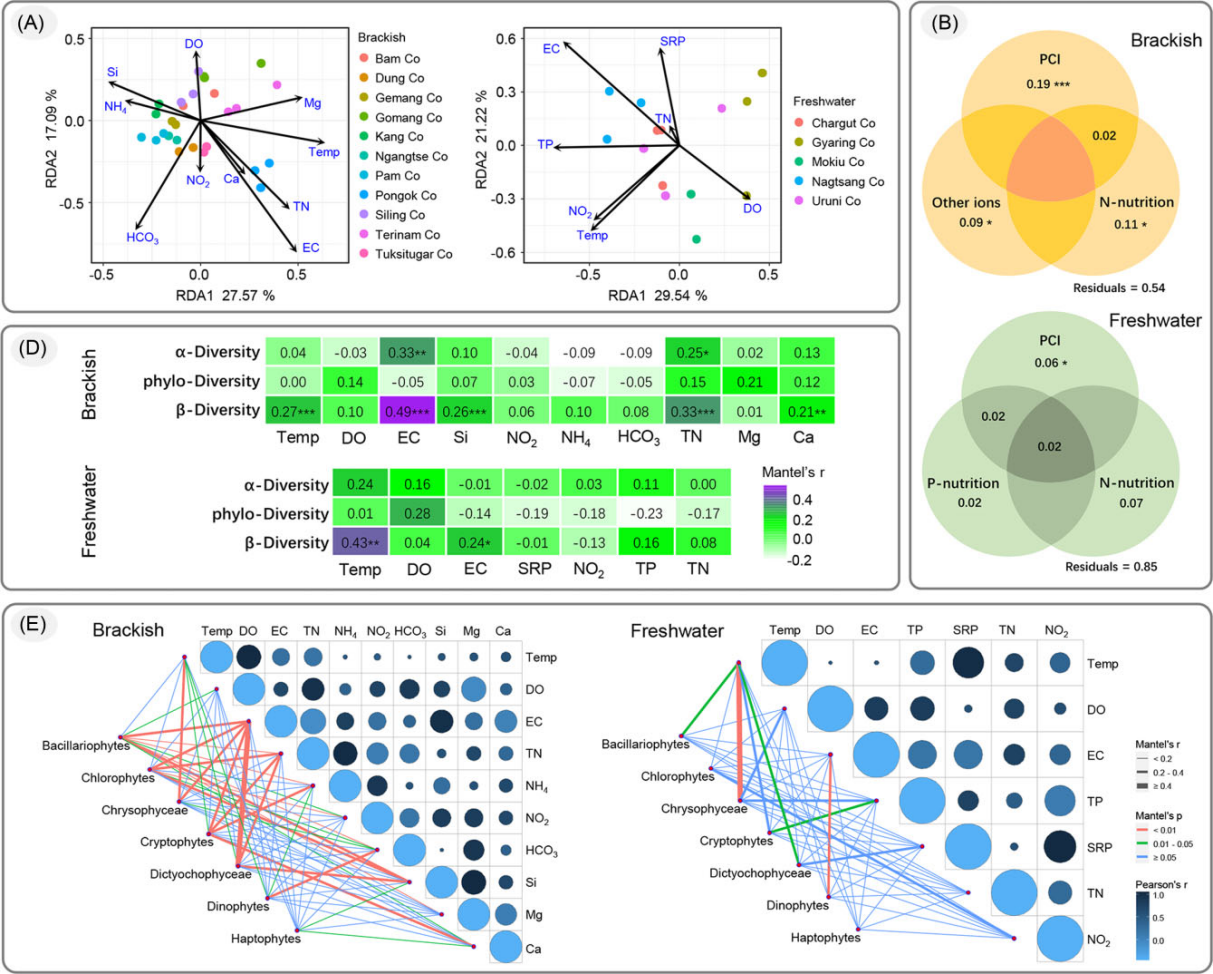
Correlation analysis between community distance and explanatory variables. (A) Biplot (samples and environmental variables) of RDA based on brackish and freshwater phytoplankton abundance matrix, respectively. (B) VPA-based Venn diagrams showing variation according to Bray–Curtis community distance explained by distinct and combined effects of PCI, phosphorus nutrition (P-nutrition), and nitrogen nutrition (N-nutrition). (C) Mantel test results of environmental variables with alpha diversity (Shannon–Wiener index), phylogenetic diversity, and beta diversity (Bray–Curtis distance), respectively. *, **, and *** represent *P* < 0.05, *P* < 0.01, and *P* < 0.001, respectively. (D) Pairwise comparisons of environmental factors with a color gradient denoting Pearson's correlation coefficient, and Mantel test results of major phytoplanktonic groups with environmental factors, the size of circle represent the correlation between environmental variables.

Following calculation of variance inflation factor (only factors with variance inflation factor <10, and Pearson coefficient <0.75 were retained), 10 and 7 environmental factors were detected in brackish and freshwater lakes, respectively (Figs S3–S4). In brackish lakes, RDA based on the abundance of phytoplankton and filtered environmental variables indicated an ordination of lakes related to physicochemical indices (PCI, such as temperature, EC, and pH) and biogenic elements (N, P, Ca, and Si). The first two axes accounted for 25.58% and 16.22% of the variance, respectively (Fig. 3A), and VPA results showed that these seven variables accounted for 46% variance, of which PCI accounted for 19% variance and nitrogenic nutrition accounted for 11% variance (Fig. 3B). In freshwater lakes, the first two axes in RDA accounted for 29.54% and 21.22% of the variance, respectively (Fig. 3A), and the phytoplankton communities in freshwater lakes clearly showed an ordination related to EC and temperature. The VPA results suggested that temperature and EC accounted for 6% variance, and nitrogenic nutrition and phosphorus nutrition accounted for 7% and 2% variance, respectively (Fig. 3B). The Mantel test results revealed no significant relationship between environmental variables and phylogenetic diversity, and only EC and TN were correlated with Shannon–Wiener diversity index in brackish lakes. The dissimilarity based on Bray-Curtis distance of phytoplankton community in brackish lakes presented significant correlation with temperature, EC, silicon, TN, and calcium (Fig. 3C). In freshwater lakes, both phylogenetic diversity and Shannon–Wiener diversity indices exhibited no significant correlation with any environmental factors. The dissimilarity of phytoplankton community showed significant correlation with temperature and EC (Fig. 3C). In summary, the results of Mantel test were closely consistent with those of RDA and VPA, thus indicating that the phylogenetic diversity of phytoplankton in central QTP lakes is independent of most of the environmental factors. However, the phytoplankton community dissimilarity was significantly correlated with PCI, especially temperature and EC, in both brackish and freshwater lakes. In particular, temperature significantly affected the dominant algal group in freshwater lakes (namely, Chrysophytes) (Fig. 3D), whereas EC and TN were the major factors influencing the dominant algal group in brackish lakes (namely, Bacillariophytes, Chlorophytes, Chrysophytes, and Dictyochophytes) (Fig. 3D).

### Threshold indicators and change points along key environmental variables gradient

Although six environmental variables (temperature, EC, TN, silicon, and calcium) were identified as significant key factors influencing phytoplankton communities in brackish lakes, only two exhibited significant change points (Table 1). A total of 5 and 6 OTUs were annotated as negative indicators across the full gradients along temperature and EC, respectively, whereas 5 and 5 OTUs were identified as positive indicators across the gradients of the above-mentioned five variables, respectively (Table 1; Figs S5–S8). There was no significant threshold indicators and change points across the five key environmental variables in freshwater lakes.

**Table 1.** Change points of key environmental variables and their responding OTUs according to TITAN results.

Variable	Change point		Responding OTUs	
	Sum(z-)	Sum(z+)	Positive	Negative
Temperature	9.7	11.3	OTU1164,OTU88,OTU25,OTU59,OTU132	OTU200,OTU349,OTU232,OTU100,OTU346
EC	13.9	17.2	OTU129,OTU525,OTU39,OTU130,OTU27,OTU17	OTU80,OTU232,OTU262,OTU206,OTU82

Overall, temperature and EC were the two most significant variables affecting phytoplankton diversity and assembly in freshwater and brackish lakes in QTP. As the daily and annual variation in water temperature in the same lake is very significant, this study mainly focused on the effects of EC gradient on phytoplankton. Only EC exhibited a gradient variation among the 16 lakes, and played a key role in shaping the phytoplankton community structure in both brackish and freshwater lakes; hence, EC was analyzed in the subsequent nCP analysis. All brackish lakes were classified into three subtypes according to the change points of each key variables, namely, pre-change point lakes (BC), inter-change point lakes (MC), and post-change point lakes (AC). All freshwater lakes were classified as one group (FW) together with the other three brackish lake types. Subsequently, the alpha diversity, beta diversity, and quantitative process of assembly of phytoplankton in all the lake types were respectively compared. The results revealed that the alpha diversity of phytoplankton in freshwater lakes was significantly higher than that in the other three brackish lakes subgroups, with no significant difference noted among the three brackish lakes subgroups (Fig. S9). The similarity of the phytoplankton communities gradually decreased with increasing EC and was the highest in MC lakes (Fig. S10), suggesting that the phytoplankton community dissimilarity exhibited a normal distribution trend along the EC, with maximum diversity noted in the EC range of about 14–17 ms/cm.

### Quantitative estimation of phytoplankton assembly process

**Figure 4.**
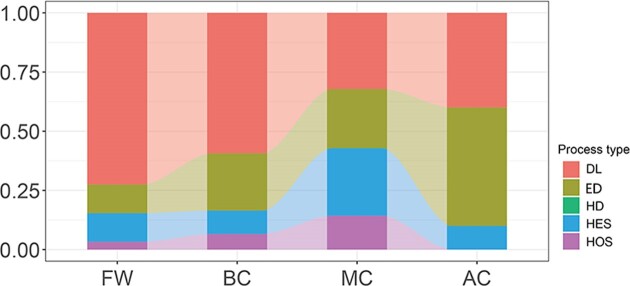
Percentage of each assembly process in each lake group. FW, freshwater lakes; BC, brackish lakes with EC < 14 ms/cm; MC, brackish lakes with EC = 14–17 ms/cm; AC, brackish lakes with EC > 17 ms/cm. DL, Dispersal limitation; ED, Ecological drift; HD, Homogenous dispersal; HES, Heterogeneous selection; HOS, Homogeneous selection.

The proportions of the four assembly processes significantly varied among the four types of lakes. Dispersal limitation was predominant in freshwater lakes, and only accounted for about 30% in brackish lakes, with no significant difference in the three types of brackish lakes (Fig. 4). The proportion of ecological drift increased with the EC, with no significant difference between BC and MC lakes; however, the value was significantly higher than that in freshwater lakes and lower than that in AC lakes (Fig. 4). The proportion of selection (including homogeneous and heterogeneous selection) was the highest in MC lakes, but was significantly lower in the other three types of lakes. Overall, it can be concluded that the ecologically neutral processes were dominant in both brackish and freshwater lakes.

## Discussion

In the present study, Dinoflagellates and Chrysophytes were the predominant groups in both alpine freshwater and brackish lakes. However, the relative abundance of Bacillariophytes was higher in freshwater lakes, while that of Cryptophytes and Chlorophytes was higher in brackish lakes. A previous study on phytoplankton assembly in coastal lakes had reported that diatoms and Cryptophytes are indicators that prefer freshwater lakes, whereas Cyanobacteria and green algae are predominant in brackish lakes (Obolewski et al. 2018). On the contrary, microscopic investigations have revealed that Bacillariophytes are the absolutely dominant phytoplankton in lakes with different salinity across the QTP (Li et al. 2021). These inconsistent results may be attributed to the huge difference in the identification methods employed. It must be noted that Uthermoll sedimentation method and microscopic counting can easily detect Bacillariophytes taxa, and may miss some fragile taxa such as Chrysophytes as well as some pico algae (with diameter less than 2 μm). Besides, these variations in results could also be attributed to the differences in the study area and elevation of the lake.

Phytoplankton diversity is known to be correlated with altitudes and coordinates, mainly owing to the changes in lake PCI and nutrient levels along with the variations in altitudes and coordinates (Kamykowski et al. 2002, Bergström and Karlsson 2019). In the present study, the investigated lakes are located in the same geographical region, under same climatic conditions and almost free from human activities. Hence, the effects of both geographic differences and human activities on phytoplankton community in these lakes were not discussed. Although EC (or salinity) can significantly distinguish lakes into freshwater and brackish, only few studies had focused on comparisons of phytoplankton in freshwater and brackish lakes, especially in alpine lakes. The species diversity, community structure, and dissimilarity of phytoplankton in these lakes significantly differ, with freshwater lakes having higher species diversity and brackish lakes having higher community dissimilarity. Previous studies have shown that phytoplankton diversity is higher in semi-brackish water than in brackish and freshwater, and this phenomenon is well explained by the intermediate disturbance hypothesis (Irena et al. 2013). In coastal reservoirs, the slight increase in salinity could decrease the specific diversity of eukaryotic phytoplankton (Mo et al. 2021). However, it must be noted that the sampling sites with different salinity employed in these studies had been in the same reservoir or lake in general. Therefore, these conclusions may not applicable to the mutually independent and unconnected lakes in central QTP.

PCI can directly or indirectly affect the metabolic activity of algae, and can thus reshape the phytoplankton community structure in general (Zohary et al. 2021). Hence, we presumed that phytoplankton community is strongly influenced by physicochemical factors (temperature, EC, etc.) in ultra-alpine freshwater lakes. It has been reported that phytoplankton communities in mountain lakes are mainly related to phosphorus, nitrogen, and silicon contents, with Bacillariophytes predominantly closely related to silicon content and green algae mainly related to salinity (Krupa and Barinova 2016), consistent with the findings of the present study in brackish lakes. In addition, salinity and EC have been noted to strongly affect phytoplankton structure and abundance in the Great Salt Lakes, QTP lakes, and Baltic Sea coastal lakes (Barrett and Belovsky 2020; Obolewski et al. 2018; Li et al. 2021), which is in agreement with the results of the present study in brackish lakes. Although both brackish and freshwater lakes are affected by PCI such as water temperature and EC, phytoplankton communities as well as their main groups in brackish and freshwater lakes have been observed to show different environmental drivers. In the present study, a significant positive correlation was found between the four algal groups (Bacillariophytes, Chlorophytes, Chrysophytes, and Dictyochophytes) and silicon concentration, which may be owing to the fact that Bacillariophytes, Dictyochophytes, Chrysophytes, and some Haptophytes form silica theca or silica scale during their life cycle (Eikrem et al. 2017, Kristiansen and Škaloud 2017, Mann et al. 2017). Furthermore, Chrysophytes, the predominant group, were affected by temperature, as most of them occur in the cold water of brackish and freshwater lakes. In the alpine freshwater lakes, pH, phosphorus, and EC have been found to be the three major variables (Grossmann et al. 2016). Besides nitrogen, phosphorus has been noted to primarily play a key role in determining the phytoplankton community of subpolar freshwater lakes (Arvola et al. 2011). It has been reported that total phosphorus, alkalinity, and water color are the major factors that influence the large-scale distribution patterns of dominant phytoplankton groups across European lakes (Maileht et al. 2013). However, the results of the present study showed that phytoplankton in QTP freshwater lakes were not affected by phosphorus or nitrogen contents, unlike those in QTP brackish lakes or freshwater lakes in other regions.

Stochastic processes were observed to govern the microbial community and regulate its assembly in alpine freshwater and brackish lakes. However, the dominance of selection or speciation in lakes with different EC showed variation. A previous study indicated that phytoplankton in QTP lakes have low alpha diversity and high beta diversity owing to the harsh environmental conditions, suggesting that assembly process is mainly dominated by probabilistic dispersal, especially dispersal limitation (Yang et al. 2018). Likewise, phytoplankton in the lakes of Inner Mongolia Plateau have also been reported to be influenced by stochastic processes (Liu et al. 2022). Similar results have also been obtained in some alpine freshwater lakes in Europe and North America (Gendron et al. 2019, Monchamp et al. 2019). The contribution of deterministic processes in eukaryotic phytoplankton community assembly increased with increasing salinity, similar to that reported by Liu et al. (2022). In addition, the proportion of deterministic processes increased with increasing EC in brackish lakes until EC >17 ms/cm, and the ecological drift and dispersal limitation were the two main mechanisms across the central QTP lakes.

**Figure 5.**
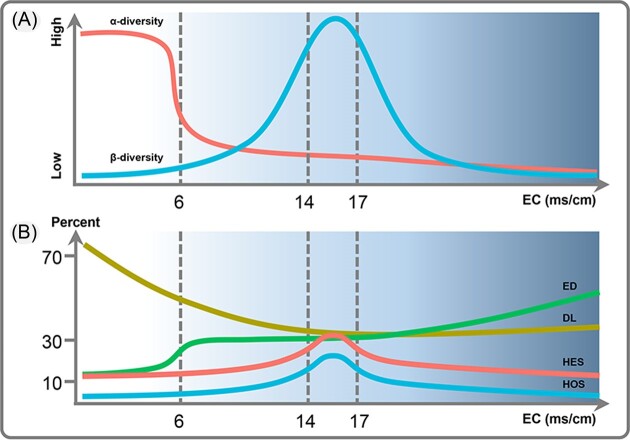
Schematic illustration of variations in the phytoplankton diversity and assembly process along EC. (A) Trends of alpha diversity (red line) and beta diversity (blue line) along EC. (B) Trends of four basal assembly processes (in %) in phytoplankton communities along EC. ED, Ecological drift; DL, Dispersal limitation; HES, Heterogeneous selection; HOS, Homogeneous selection.

The phytoplankton diversity and structure are known to show regular shifts along with changes in different environmental factors (Vallina et al. 2017). However, it is difficult to explore the underlying mechanism of these environmental variables and determine their change points (Edwards et al. 2013). The findings of the present study showed that phytoplankton in both freshwater and brackish lakes in the central QTP are significantly affected by EC, especially in lakes with EC around 6, 14, and 17 ms/cm. The TITAN results revealed no significant change thresholds and representative indicator in freshwater lakes. Thus, we hypothesized that there may not be any significant differences in phytoplankton community changes in freshwater lakes, and that the impact of climate change on freshwater lakes may be relatively small. However, our results showed that the decreased EC did not lead to significant changes in the phytoplankton species diversity in most of the brackish lakes, except in lakes with EC ≤ 6 ms/cm, whereas an obvious increase in species diversity was noted in the semi-brackish lakes. In lakes with EC of around 14 ms/cm, a decrease in EC and a subsequent increase to around 17 ms/cm caused a decrease in phytoplankton community dissimilarity. Such obvious shifts in phytoplankton diversity and structure are schematically summarized in Fig. 5A. Moreover, a gradual decrease in EC also affected the phytoplankton assembly process, and species dispersal limitation progressively increased in semi-brackish lakes. In lakes with EC of around 14 ms/cm, the proportion of selection decreased. Overall, the effect of EC on the phytoplankton assembly process in alpine lakes mainly regulated the proportion of specific process, while both freshwater and brackish lakes were generally dominated by stochastic processes (Fig. 5B).

## Conclusion

The present study revealed that EC (or salinity) is powerful structuring force on phytoplankton structuring and assembly process in central QTP lakes. The phytoplankton in these lakes could be extensively classified into freshwater and brackish types, with significant difference in species and community dissimilarity between them. Although phytoplankton communities in both freshwater and brackish lakes were significantly affected by EC and temperature, brackish phytoplankton were also significantly influenced by TN, silicon, and calcium contents. Stochastic processes were dominant in central QTP lakes, with the proportion of species dispersal limitation gradually decreasing and ecological drift gradually increasing with the increasing EC. Significant differences in EC gradients were noted in brackish lakes, and these lakes could be distinguished into three types based on EC, with an EC threshold of about 14–17 ms/cm. The phytoplankton species diversity, community dissimilarity, and assembly process showed obvious differences across this EC threshold. Thus, these results suggest that the decrease in lake EC due to warming and moistening from climate change may have almost no effect on phytoplankton in freshwater lakes, but could afftect phytoplankton in brackish lakes, particularly those with an EC of around 14–17 ms/cm. Overall, the findings reveal that the effects of lake desalination on eukaryotic phytoplankton depend on the EC regime, and provide a mechanistic basis for understanding global climate change in Qinghai-Tibet Plateau aquatic ecosystems.

## Supplementary Material

fiad163_Supplemental_Files
