# Supplementary material for: Lakes-scale pattern of eukaryotic phytoplankton diversity and assembly process shaped by electrical conductivity in central Qinghai-Tibet Plateau
Source: FEMS Microbiol Ecol. 2023 Dec 14;100(1):fiad163. doi: 10.1093/femsec/fiad163 (PMC10791044; doi:10.1093/femsec/fiad163)
Supplement: fiad163_Supplemental_Files [file fiad163_supplemental_files.zip › supplemental data figures.pdf]

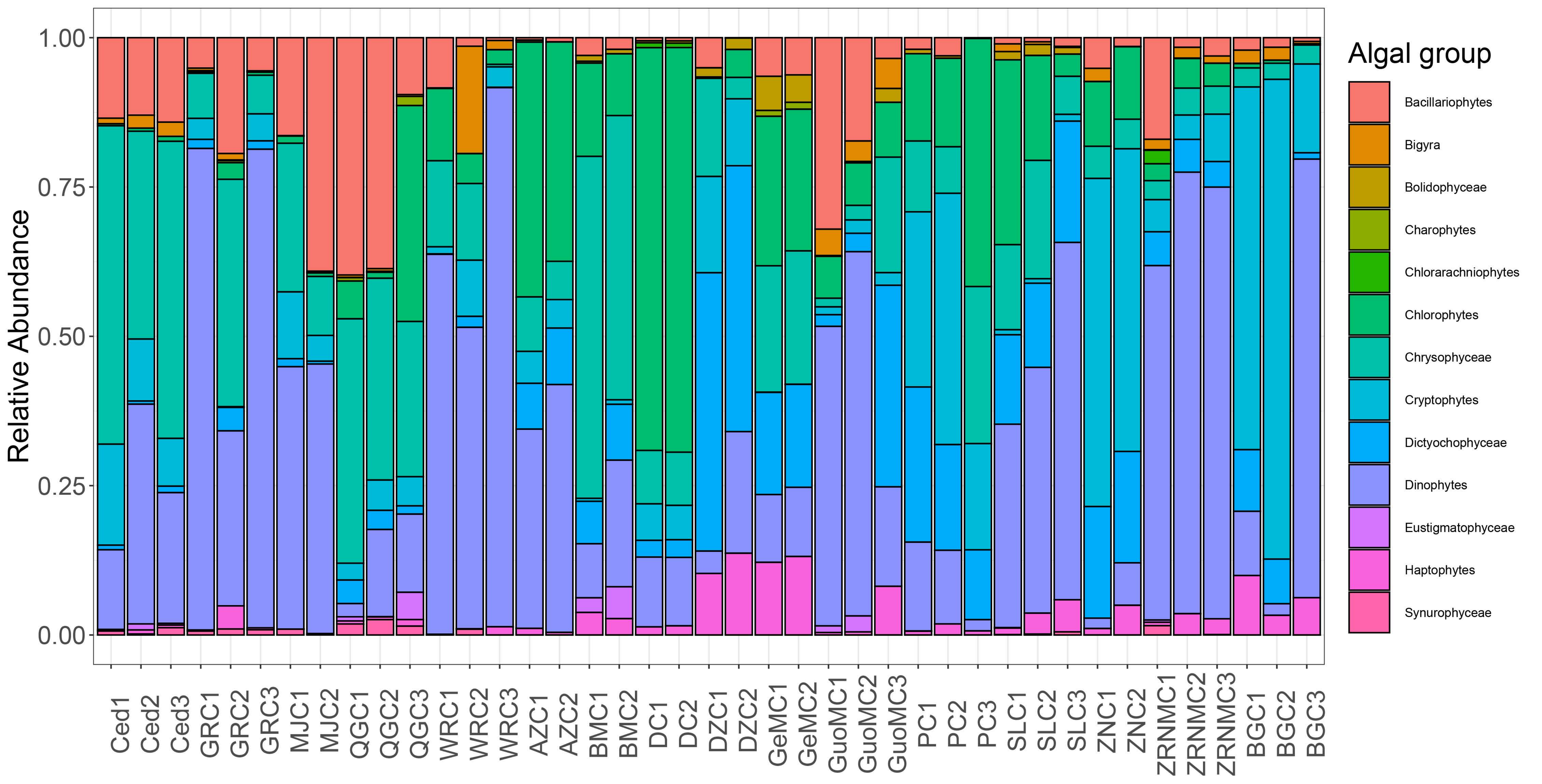
Relative Abundance
1.00
0.75
0.50
0.25
0.00
Ced1
Ced2
Ced3
GRC1
GRC2
GRC3
MJC1
MJC2
QGC1
QGC2
QGC3
WRC1
WRC2
WRC3
AZC1
AZC2
BMC1
BMC2
DC1
DC2
DZC1
DZC2
GeMC1
GeMC2
GuoMC1
GuoMC2
GuoMC3
PC1
PC2
PC3
SLC1
SLC2
SLC3
ZNC1
ZNC2
ZRNMC1
ZRNMC2
ZRNMC3
BGC1
BGC2
BGC3
Algal group
Bacillariophytes
Bigyra
Bolidophyceae
Charophytes
Chlorarachniophytes
Chlorophytes
Chrysophyceae
Cryptophytes
Dictyochophyceae
Dinophytes
Eustigmatophyceae
Haptophytes
Synurophyceae

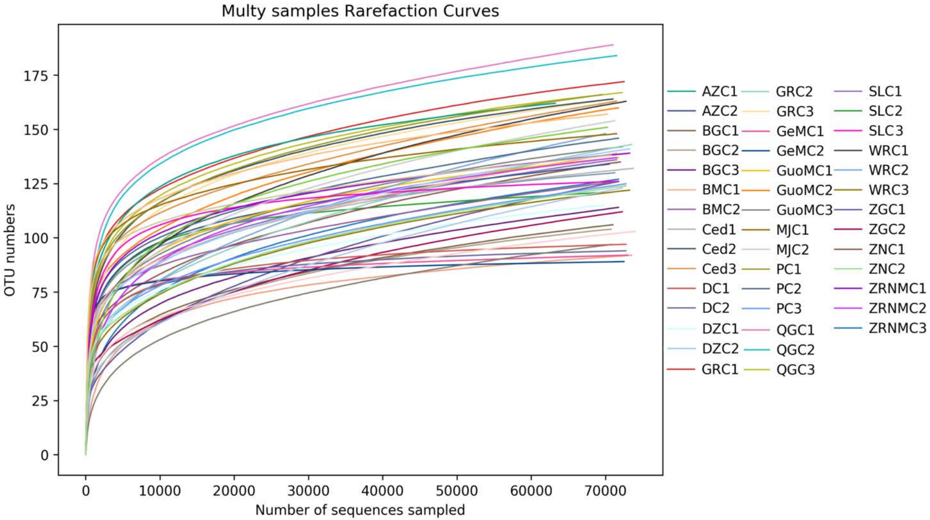
Multy samples Rarefaction Curves
OTU numbers
Number of sequences sampled
0
25
50
75
100
125
150
175
0
10000
20000
30000
40000
50000
60000
70000
AZC1
AZC2
BGC1
BGC2
BGC3
BMC1
BMC2
Ced1
Ced2
Ced3
DC1
DC2
DZC1
DZC2
GRC1
GRC2
GRC3
GeMC1
GeMC2
GuoMC1
GuoMC2
GuoMC3
MJC1
MJC2
PC1
PC2
PC3
QGC1
QGC2
QGC3
SLC1
SLC2
SLC3
WRC1
WRC2
WRC3
ZGC1
ZGC2
ZNC1
ZNC2
ZRNMC1
ZRNMC2
ZRNMC3

	Temp	DO	pH	EC	TDS	Sal	SRP	Si	NO2	NH4	TAlk	CO3	HCO3	TN	TOC	COD	TP	NO3	Cl	SO4	Na	K	Mg	Ca
Temp	1	-0.61	-0.24	0.27	0.28	0.27	0.43	-0.08	0.07	-0.02	0.16	0.15	0	0.3	0.25	0.23	0.49	0.28	0.24	0.03	0.2	0.32	-0.09	-0.1
DO	-0.61	1	-0.07	-0.21	-0.21	-0.21	-0.36	-0.24	-0.24	0.04	-0.31	-0.23	-0.37	-0.49	-0.51	-0.42	-0.48	-0.53	-0.14	-0.07	-0.2	-0.2	0.59	0.14
pH	-0.24	-0.07	1	0.08	0.06	0.06	0.09	0.07	0.34	-0.09	0.78	0.75	0.54	0.06	0.13	0.07	0.14	0.04	-0.29	-0.01	0.13	-0.02	-0.27	-0.22
EC	0.27	-0.21	0.08	1	1	1	0.82	-0.51	0.31	-0.32	0.62	0.73	0.16	0.63	0.53	0.39	0.82	0.56	0.84	0.88	0.91	0.96	0.29	0.5
TDS	0.28	-0.21	0.06	1	1	1	0.83	-0.51	0.3	-0.31	0.62	0.72	0.16	0.64	0.53	0.4	0.83	0.57	0.84	0.89	0.91	0.96	0.29	0.5
Sal	0.27	-0.21	0.06	1	1	1	0.83	-0.51	0.3	-0.31	0.62	0.72	0.16	0.64	0.53	0.4	0.83	0.57	0.84	0.89	0.91	0.96	0.29	0.5
SRP	0.43	-0.36	0.09	0.82	0.83	0.83	1	-0.11	0.33	-0.26	0.58	0.64	0.23	0.69	0.62	0.49	0.95	0.65	0.69	0.67	0.75	0.78	0	0.3
Si	-0.08	-0.24	0.07	-0.51	-0.51	-0.51	-0.11	1	-0.27	0.33	-0.24	-0.33	0.1	-0.09	0.04	-0.03	-0.15	0.03	-0.44	-0.44	-0.41	-0.57	-0.64	-0.24
NO2	0.07	-0.24	0.34	0.31	0.3	0.3	0.33	-0.27	1	-0.36	0.39	0.37	0.36	0.45	0.39	0.45	0.39	0.41	0.22	0.37	0.37	0.22	-0.34	-0.19
NH4	-0.02	0.04	-0.09	-0.32	-0.31	-0.31	-0.26	0.33	-0.36	1	-0.16	-0.23	-0.02	-0.52	-0.52	-0.6	-0.37	-0.52	-0.5	-0.3	-0.23	-0.25	-0.21	-0.26
TAlk	0.16	-0.31	0.78	0.62	0.62	0.62	0.58	-0.24	0.39	-0.16	1	0.97	0.74	0.44	0.42	0.34	0.63	0.39	0.16	0.46	0.64	0.58	-0.06	0.18
CO3	0.15	-0.23	0.75	0.73	0.72	0.72	0.64	-0.33	0.37	-0.23	0.97	1	0.57	0.43	0.4	0.29	0.67	0.37	0.3	0.55	0.7	0.68	0.07	0.22
HCO3	0	-0.37	0.54	0.16	0.16	0.16	0.23	0.1	0.36	-0.02	0.74	0.57	1	0.36	0.39	0.41	0.29	0.38	-0.21	0.14	0.3	0.13	-0.43	0.11
TN	0.3	-0.49	0.06	0.63	0.64	0.64	0.69	-0.09	0.45	-0.52	0.44	0.43	0.36	1	0.95	0.87	0.79	0.96	0.68	0.62	0.64	0.54	-0.2	0.2
TOC	0.25	-0.51	0.13	0.53	0.53	0.53	0.62	0.04	0.39	-0.52	0.42	0.4	0.39	0.95	1	0.93	0.75	0.98	0.61	0.54	0.56	0.42	-0.29	0.15
COD	0.23	-0.42	0.07	0.39	0.4	0.4	0.49	-0.03	0.45	-0.6	0.34	0.29	0.41	0.87	0.93	1	0.64	0.92	0.51	0.45	0.42	0.3	-0.27	0.09
TP	0.49	-0.48	0.14	0.82	0.83	0.83	0.95	-0.15	0.39	-0.37	0.63	0.67	0.29	0.79	0.75	0.64	1	0.76	0.71	0.67	0.77	0.75	-0.09	0.22
NO3	0.28	-0.53	0.04	0.56	0.57	0.57	0.65	0.03	0.41	-0.52	0.39	0.37	0.38	0.96	0.98	0.92	0.76	1	0.65	0.59	0.6	0.46	-0.3	0.24
Cl	0.24	-0.14	-0.29	0.84	0.84	0.84	0.69	-0.44	0.22	-0.5	0.16	0.3	-0.21	0.68	0.61	0.51	0.71	0.65	1	0.82	0.72	0.78	0.32	0.47
SO4	0.03	-0.07	-0.01	0.88	0.89	0.89	0.67	-0.44	0.37	-0.3	0.46	0.55	0.14	0.62	0.54	0.45	0.67	0.59	0.82	1	0.86	0.81	0.22	0.49
Na	0.2	-0.2	0.13	0.91	0.91	0.91	0.75	-0.41	0.37	-0.23	0.64	0.7	0.3	0.64	0.56	0.42	0.77	0.6	0.72	0.86	1	0.78	0.1	0.5
K	0.32	-0.2	-0.02	0.96	0.96	0.96	0.78	-0.57	0.22	-0.25	0.58	0.68	0.13	0.54	0.42	0.3	0.75	0.46	0.78	0.81	0.78	1	0.41	0.49
Mg	-0.09	0.59	-0.27	0.29	0.29	0.29	0	-0.64	-0.34	-0.21	-0.06	0.07	-0.43	-0.2	-0.29	-0.27	-0.09	-0.3	0.32	0.22	0.1	0.41	1	0.46
Ca	-0.1	0.14	-0.22	0.5	0.5	0.5	0.3	-0.24	-0.19	-0.26	0.18	0.22	0.11	0.2	0.15	0.09	0.22	0.24	0.47	0.49	0.5	0.49	0.46	1

1, 0.8, 0.6, 0.4, 0.2, 0, -0.2, -0.4, -0.6, -0.8, -1

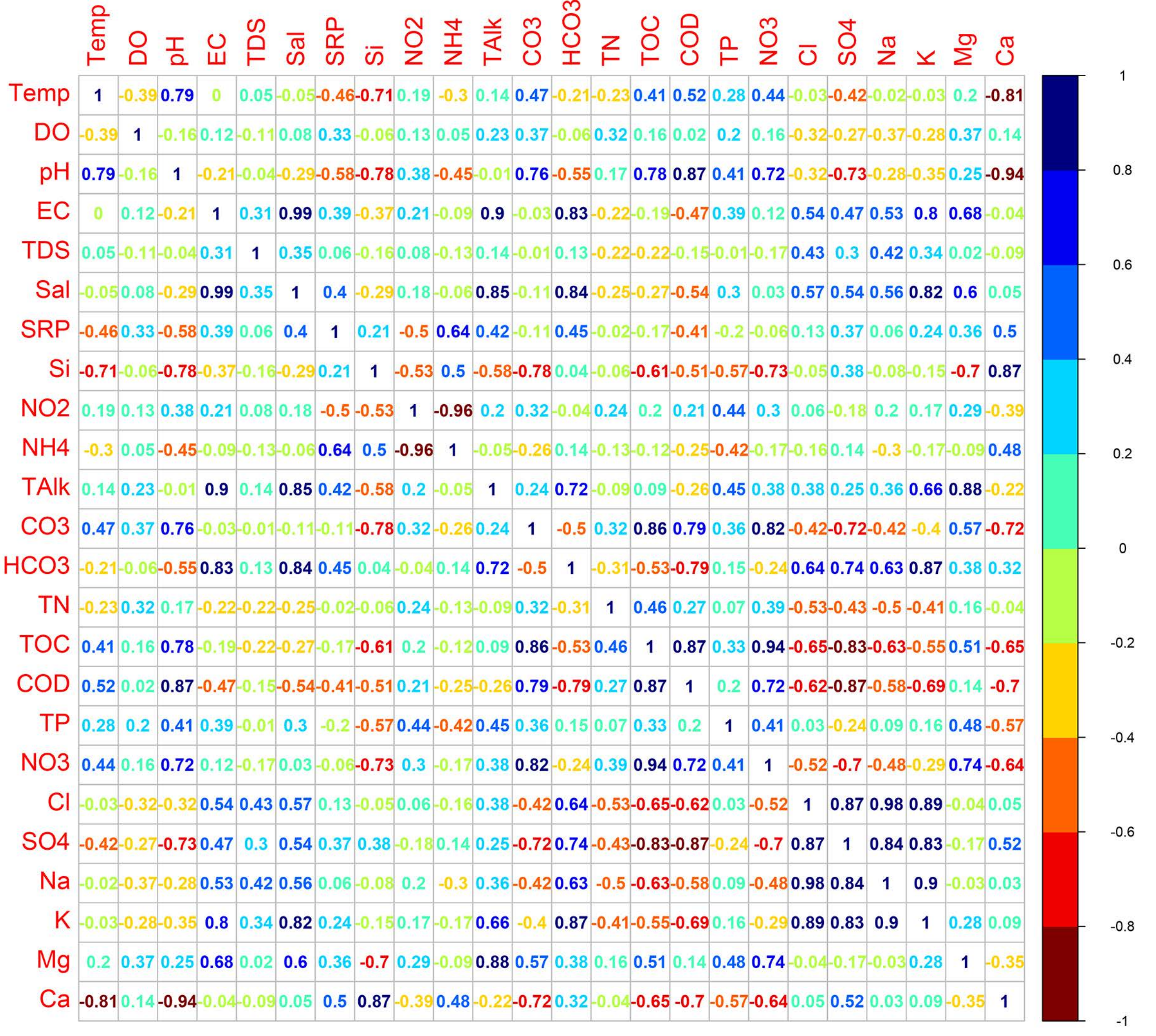
Temp DO pH EC TDS Sal SRP Si NO2 NH4 TAlk CO3 HCO3 TN TOC COD TP NO3 Cl SO4 Na K Mg Ca
Temp 1 -0.39 0.79 0 0.05 -0.05 -0.46 -0.71 0.19 -0.3 0.14 0.47 -0.21 -0.23 0.41 0.52 0.28 0.44 -0.03 -0.42 -0.02 -0.03 0.2 -0.81
DO -0.39 1 -0.16 0.12 -0.11 0.08 0.33 -0.06 0.13 0.05 0.23 0.37 -0.06 0.32 0.16 0.02 0.2 0.16 -0.32 -0.27 -0.37 -0.28 0.37 0.14
pH 0.79 -0.16 1 -0.21 -0.04 -0.29 -0.58 -0.78 0.38 -0.45 -0.01 0.76 -0.55 0.17 0.78 0.87 0.41 0.72 -0.32 -0.73 -0.28 -0.35 0.25 -0.94
EC 0 0.12 -0.21 1 0.31 0.99 0.39 -0.37 0.21 -0.09 0.9 -0.03 0.83 -0.22 -0.19 -0.47 0.39 0.12 0.54 0.47 0.53 0.8 0.68 -0.04
TDS 0.05 -0.11 -0.04 0.31 1 0.35 0.06 -0.16 0.08 -0.13 0.14 -0.01 0.13 -0.22 -0.22 -0.15 -0.01 -0.17 0.43 0.3 0.42 0.34 0.02 -0.09
Sal -0.05 0.08 -0.29 0.99 0.35 1 0.4 -0.29 0.18 -0.06 0.85 -0.11 0.84 -0.25 -0.27 -0.54 0.3 0.03 0.57 0.54 0.56 0.82 0.6 0.05
SRP -0.46 0.33 -0.58 0.39 0.06 0.4 1 0.21 -0.5 0.64 0.42 -0.11 0.45 -0.02 -0.17 -0.41 -0.2 -0.06 0.13 0.37 0.06 0.24 0.36 0.5
Si -0.71 -0.06 -0.78 -0.37 -0.16 -0.29 0.21 1 -0.53 0.5 -0.58 -0.78 0.04 -0.06 -0.61 -0.51 -0.57 -0.73 -0.05 0.38 -0.08 -0.15 -0.7 0.87
NO2 0.19 0.13 0.38 0.21 0.08 0.18 -0.5 -0.53 1 -0.96 0.2 0.32 -0.04 0.24 0.2 0.21 0.44 0.3 0.06 -0.18 0.2 0.17 0.29 -0.39
NH4 -0.3 0.05 -0.45 -0.09 -0.13 -0.06 0.64 0.5 -0.96 1 -0.05 -0.26 0.14 -0.13 -0.12 -0.25 -0.42 -0.17 -0.16 0.14 -0.3 -0.17 -0.09 0.48
TAlk 0.14 0.23 -0.01 0.9 0.14 0.85 0.42 -0.58 0.2 -0.05 1 0.24 0.72 -0.09 0.09 -0.26 0.45 0.38 0.38 0.25 0.36 0.66 0.88 -0.22
CO3 0.47 0.37 0.76 -0.03 -0.01 -0.11 -0.11 -0.78 0.32 -0.26 0.24 1 -0.5 0.32 0.86 0.79 0.36 0.82 -0.42 -0.72 -0.42 -0.4 0.57 -0.72
HCO3 -0.21 -0.06 -0.55 0.83 0.13 0.84 0.45 0.04 -0.04 0.14 0.72 -0.5 1 -0.31 -0.53 -0.79 0.15 -0.24 0.64 0.74 0.63 0.87 0.38 0.32
TN -0.23 0.32 0.17 -0.22 -0.22 -0.25 -0.02 -0.06 0.24 -0.13 -0.09 0.32 -0.31 1 0.46 0.27 0.07 0.39 -0.53 -0.43 -0.5 -0.41 0.16 -0.04
TOC 0.41 0.16 0.78 -0.19 -0.22 -0.27 -0.17 -0.61 0.2 -0.12 0.09 0.86 -0.53 0.46 1 0.87 0.33 0.94 -0.65 -0.83 -0.63 -0.55 0.51 -0.65
COD 0.52 0.02 0.87 -0.47 -0.15 -0.54 -0.41 -0.51 0.21 -0.25 -0.26 0.79 -0.79 0.27 0.87 1 0.2 0.72 -0.62 -0.87 -0.58 -0.69 0.14 -0.7
TP 0.28 0.2 0.41 0.39 -0.01 0.3 -0.2 -0.57 0.44 -0.42 0.45 0.36 0.15 0.07 0.33 0.2 1 0.41 0.03 -0.24 0.09 0.16 0.48 -0.57
NO3 0.44 0.16 0.72 0.12 -0.17 0.03 -0.06 -0.73 0.3 -0.17 0.38 0.82 -0.24 0.39 0.94 0.72 0.41 1 -0.52 -0.7 -0.48 -0.29 0.74 -0.64
Cl -0.03 -0.32 -0.32 0.54 0.43 0.57 0.13 -0.05 0.06 -0.16 0.38 -0.42 0.64 -0.53 -0.65 -0.62 0.03 -0.52 1 0.87 0.98 0.89 -0.04 0.05
SO4 -0.42 -0.27 -0.73 0.47 0.3 0.54 0.37 0.38 -0.18 0.14 0.25 -0.72 0.74 -0.43 -0.83 -0.87 -0.24 -0.7 0.87 1 0.84 0.83 -0.17 0.52
Na -0.02 -0.37 -0.28 0.53 0.42 0.56 0.06 -0.08 0.2 -0.3 0.36 -0.42 0.63 -0.5 -0.63 -0.58 0.09 -0.48 0.98 0.84 1 0.9 -0.03 0.03
K -0.03 -0.28 -0.35 0.8 0.34 0.82 0.24 -0.15 0.17 -0.17 0.66 -0.4 0.87 -0.41 -0.55 -0.69 0.16 -0.29 0.89 0.83 0.9 1 0.28 0.09
Mg 0.2 0.37 0.25 0.68 0.02 0.6 0.36 -0.7 0.29 -0.09 0.88 0.57 0.38 0.16 0.51 0.14 0.48 0.74 -0.04 -0.17 -0.03 0.28 1 -0.35
Ca -0.81 0.14 -0.94 -0.04 -0.09 0.05 0.5 0.87 -0.39 0.48 -0.22 -0.72 0.32 -0.04 -0.65 -0.7 -0.57 -0.64 0.05 0.52 0.03 0.09 -0.35 1
1
0.8
0.6
0.4
0.2
0
-0.2
-0.4
-0.6
-0.8
-1

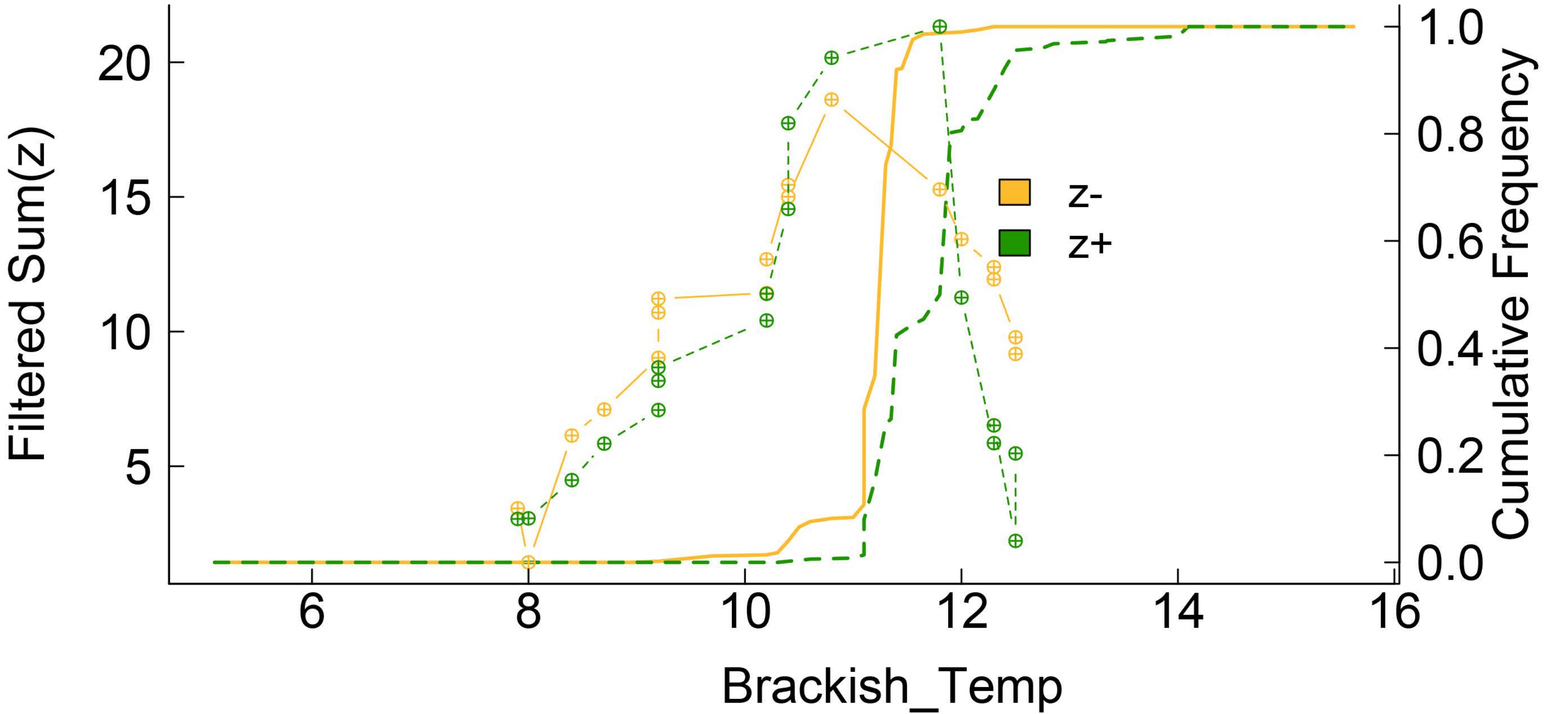

Filtered Sum(z)
Cumulative Frequency
Brackish_Temp
z-
z+
20
15
10
5
6
8
10
12
14
16
1.0
0.8
0.6
0.4
0.2
0.0

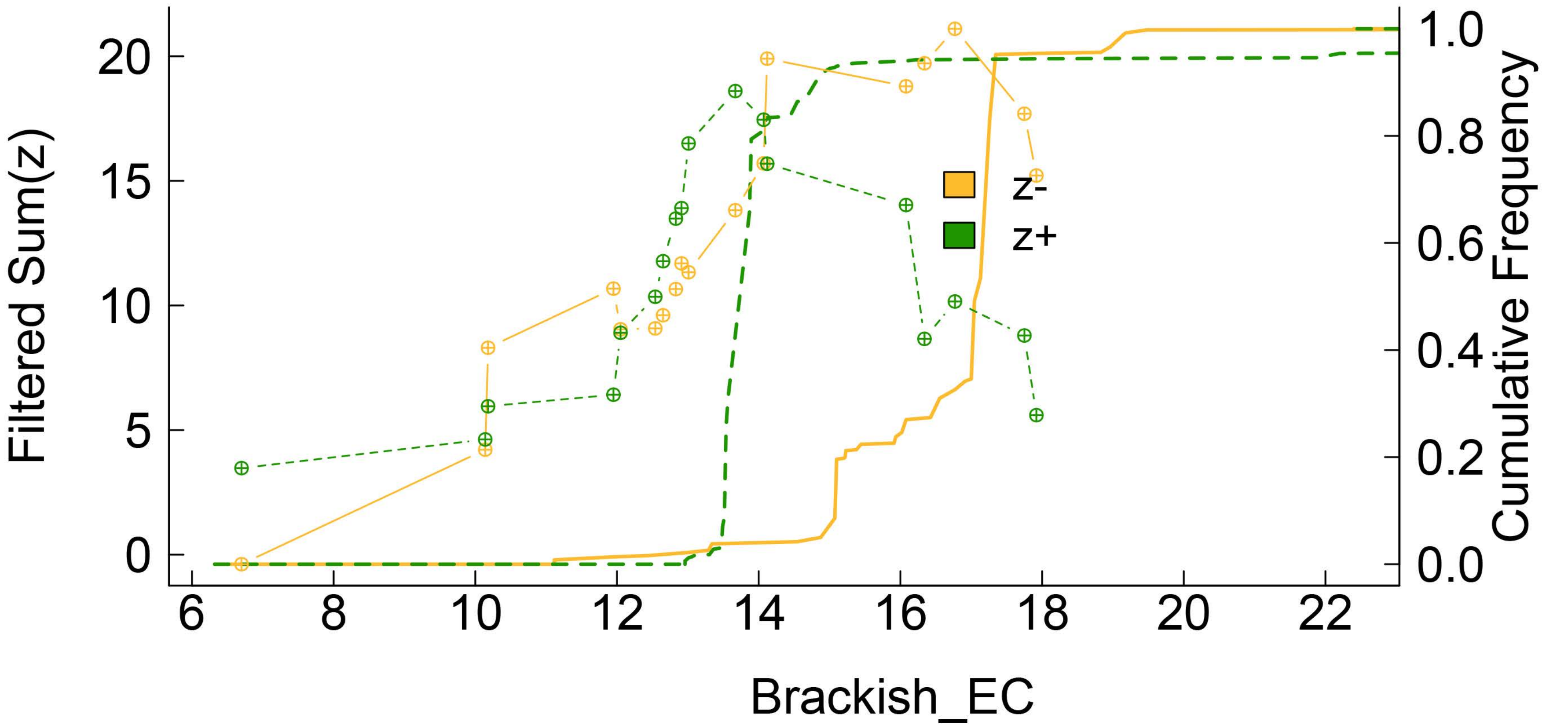

Filtered Sum(z)
Cumulative Frequency
Brackish_EC
z-
z+
0
5
10
15
20
0.0
0.2
0.4
0.6
0.8
1.0
6
8
10
12
14
16
18
20
22

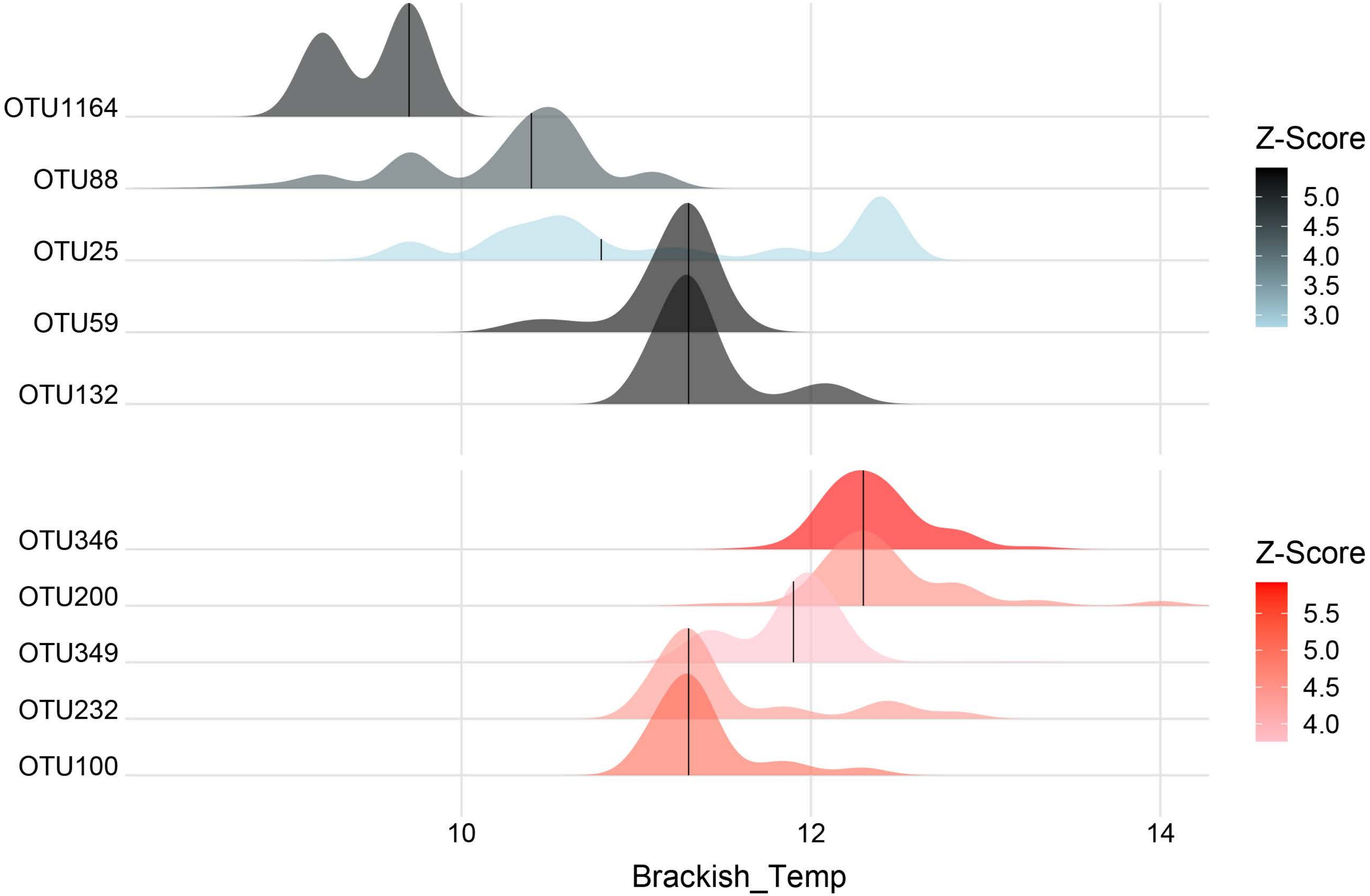
OTU1164
OTU88
OTU25
OTU59
OTU132
OTU346
OTU200
OTU349
OTU232
OTU100
Z-Score
5.0
4.5
4.0
3.5
3.0
Z-Score
5.5
5.0
4.5
4.0
10
12
14
Brackish_Temp

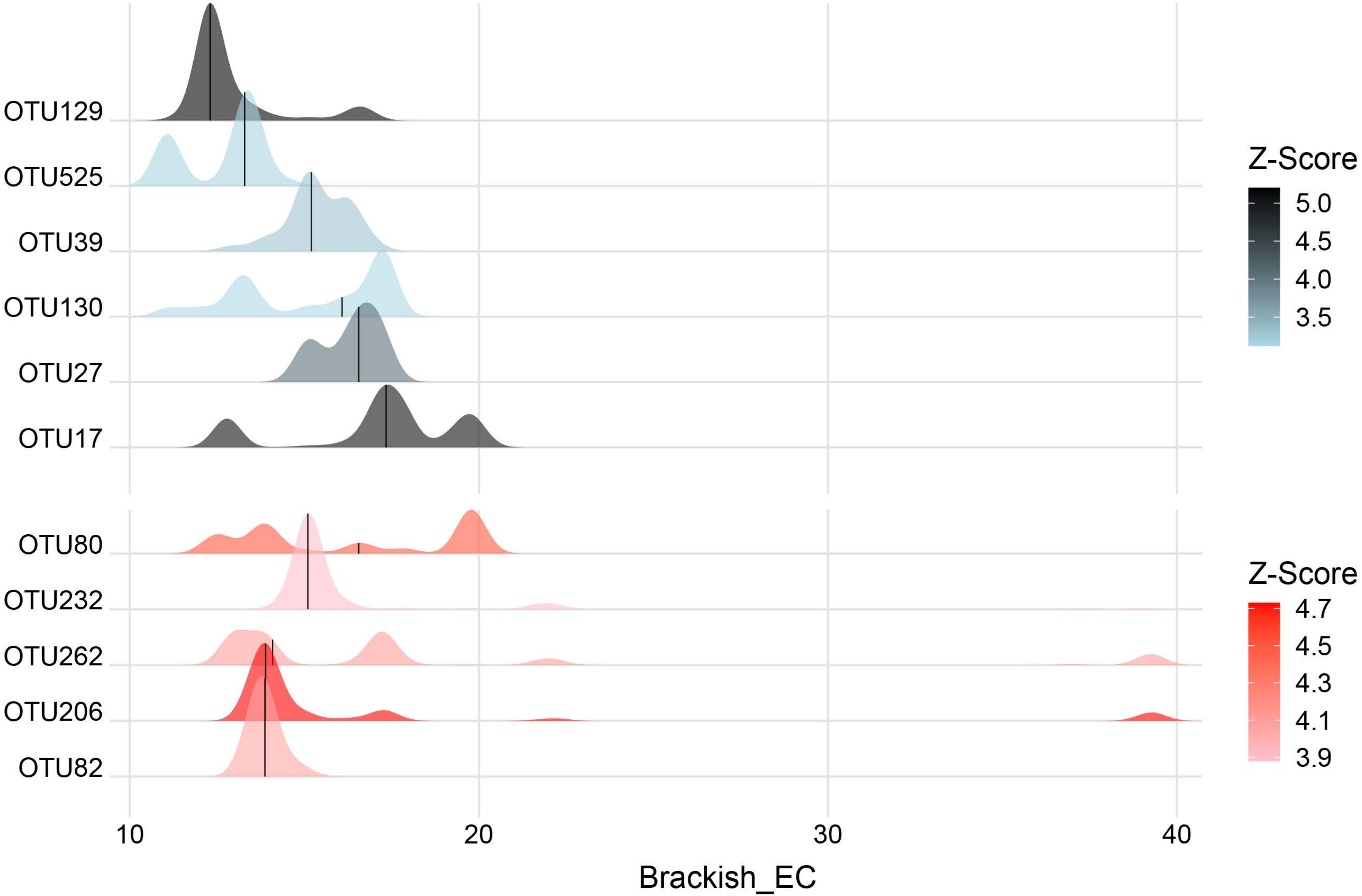
OTU129
OTU525
OTU39
OTU130
OTU27
OTU17
OTU80
OTU232
OTU262
OTU206
OTU82
Z-Score
5.0
4.5
4.0
3.5
Z-Score
4.7
4.5
4.3
4.1
3.9
10
20
30
40
Brackish_EC

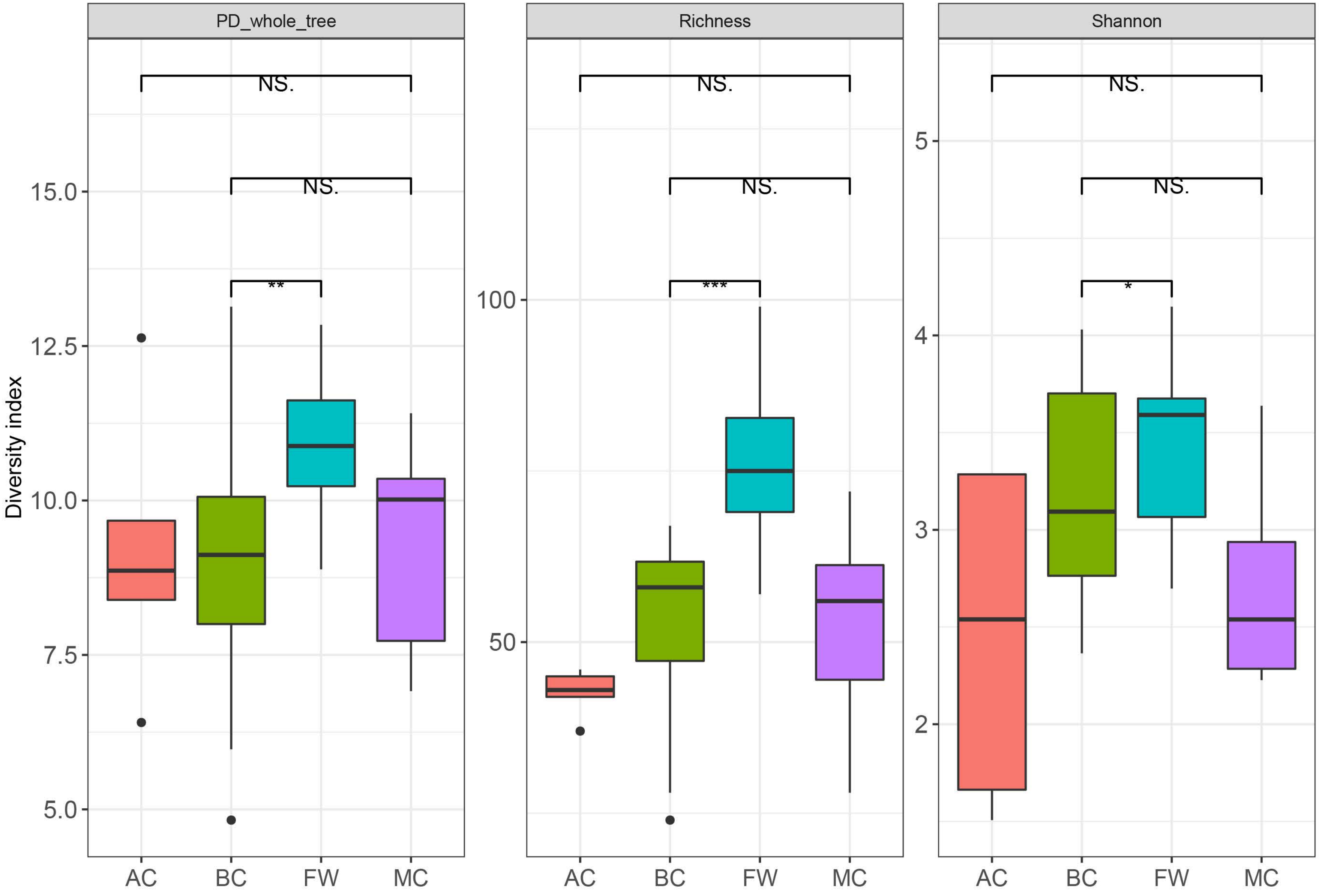

PD_whole_tree
Richness
Shannon
Diversity index
NS.
NS.
**
***
*
15.0
12.5
10.0
7.5
5.0
100
50
5
4
3
2
AC
BC
FW
MC

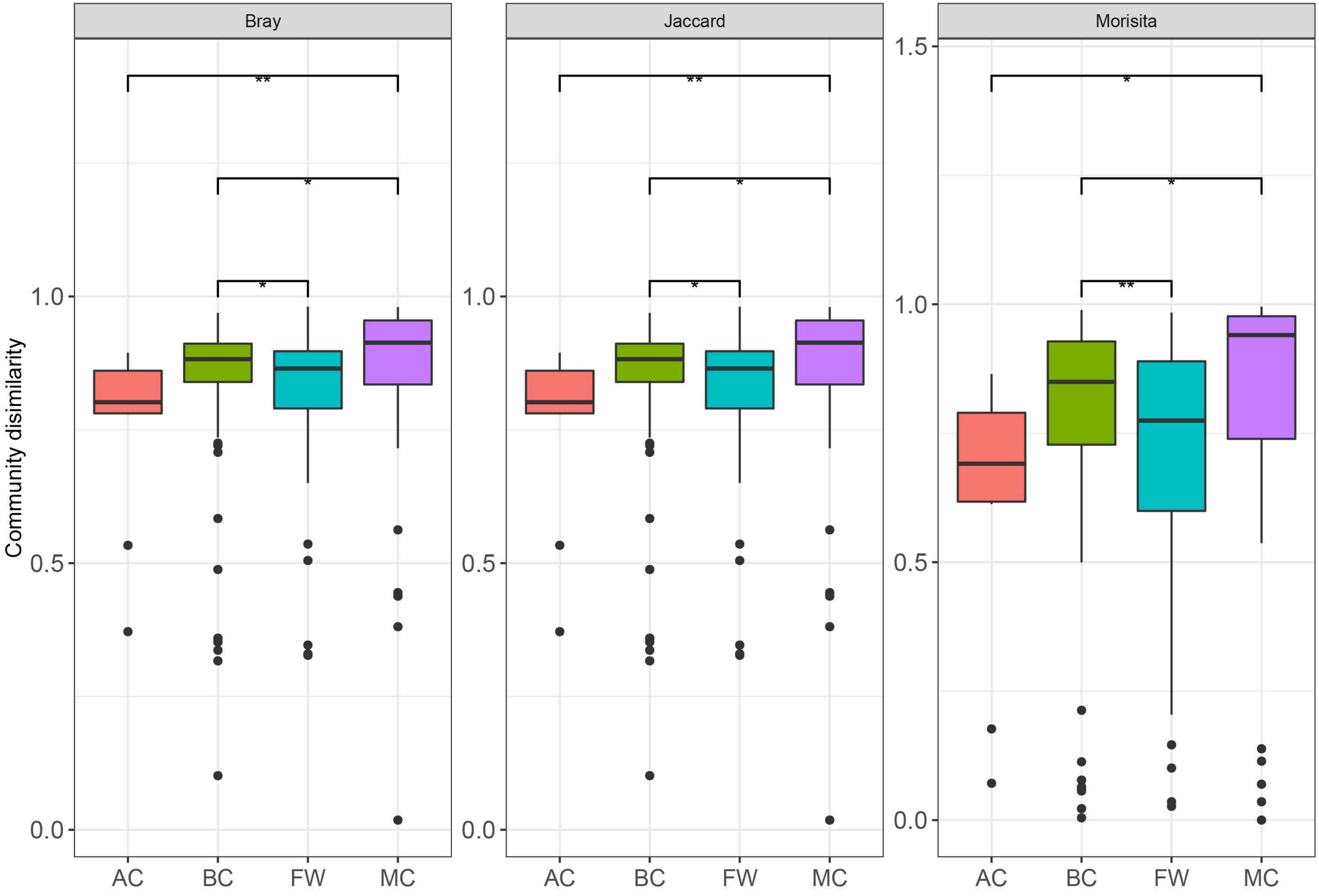

Bray
Jaccard
Morisita
Community disimilarity
0.0
0.5
1.0
1.5
**
*
AC
BC
FW
MC
